# Classifying behaviors from animal-borne cameras using machine learning: automated identification of breathing events in sea turtles

**DOI:** 10.1242/jeb.251688

**Published:** 2026-06-10

**Authors:** Nathan J. Robinson, Priyam Mazumdar, Brian F. Allan, Jacopo Aguzzi, Brian Chen, Bing-Yuan Hsieh, Lola Millet, Jesus Tomás, Jeffrey Terstriep, Aiman Soliman

**Affiliations:** ^1^ Institut de Ciències del Mar, Spanish National Research Council - Consejo Superior de Investigaciones Científicas, 08003 Barcelona, Spain; ^2^ Cape Eleuthera Institute, Cape Eleuthera Island School, Eleuthera, Bahamas; ^3^ Fundación Oceanogràfic de la Comunitat Valenciana, Ciudad de las Artes y las Ciencias, 46013 Valencia, Spain; ^4^ National Center for Supercomputing Applications, University of Illinois Urbana-Champaign, Champaign, IL 61801, USA; ^5^ Instituto Cavanilles de Biodiversidad y Biología Evolutiva, University of Valencia, Apdo, 22085, 46071 Valencia, Spain

**Keywords:** Artificial intelligence, Diving, Behavioral classification, Image processing, Machine learning, *Chelonia mydas*

## Abstract

Animal-borne cameras are increasingly used to study animal behavior. Here, we assessed the utility of three machine-learning models – Resnet-50 (3 epochs), Resnet-50 (10 epochs) and Vision Transformer (ViT) (3 epochs) – for identifying breathing behavior from animal-borne camera footage from green turtles (*Chelonia mydas*). The ViT model had mean Accuracy (97.2%), Precision (63.3%) and F1 (72.7%) scores that outperformed the Resnet-50 models, while all models had a Recall of >99.9%. Thus, the ViT model correctly identified almost all breathing frames although false positives (apnea frames labeled as breathing) were relatively common and led to an over-estimation of breathing rates. We conclude that ViT models are a promising solution for behavioral classification of animal-borne camera footage and even if not yet capable of the fully automated calculation of breathing events in sea turtles, they can still massively reduce the quantity of footage that needs to be manually checked and labeled.

## Teaser

**Summary:** Demonstration that the Vision Transformer (ViT) tool is suitable for annotating breathing behavior from animal-borne cameras deployed on sea turtles.

## INTRODUCTION

Incorporating cameras into animal-borne devices facilitated a major leap forward in the study of marine megafauna such as sea turtles, marine mammals and sharks (Hays, 2015; Wilmers et al., 2015). With the capacity to gain insights into an animal's visual surroundings (Umwelt), these devices can reveal how animals both perceive and interact with their environment (e.g. Ryan et al., 2022). However, as the use of animal-borne cameras is rapidly increasing (e.g. Thomson et al., 2011; Handley et al., 2018; Agabiti et al., 2026) and innovation in technology allows for longer recording durations, ecologists are faced with the issue of how to efficiently analyze growing quantities of footage (Hampton et al., 2013; Farley et al., 2018). One solution to this problem is to use machine-learning tools, where pre-annotated datasets are used to train pattern-identifying algorithms to annotate other datasets without the requirement for human input (Valletta et al., 2017; Tuia et al., 2022).

Animal-borne camera footage is primarily used to study the behavior of the tagged individuals (Moll et al., 2007). In the context of developing machine-learning tools for behavioral classification, a practical starting point would therefore be to identify an ecologically relevant behavior that is repeatedly and reliably observed across several different taxa. One such example from air-breathing marine megafauna, such as sea turtles, could be breathing behavior. Indeed, information on sea turtle breathing patterns can provide insights into their diving physiology (Robinson et al., 2021), foraging strategies (Okuyama et al., 2012), habitat use (Shillinger et al., 2011) and stress levels (Robinson et al., 2024). Breathing patterns for sea turtles, however, are typically inferred via time–depth recorders (TDRs) by assuming that turtles are breathing whenever they ascend above a specified depth (typically 2 m; e.g. Shillinger et al., 2011, Robinson et al., 2021). Yet, turtles at the surface may not always be breathing (Hochscheid et al., 2010; Agabiti et al., 2026) and this technique is of little use in habitats that are shallower that typically utilized depth limits (e.g. Doñate-Ordóñez et al., 2025). Similarly, efforts to estimate breathing events from accelerometers have had only limited success because of the high signal to noise ratio in the data alongside issues associated with the influence of both waves and the turtles' variable posture when breathing (Jeantet et al., 2024; Agabiti et al., 2026).

The most common machine-learning tools used for image processing and feature extraction are currently convolutional neural networks (CNN) (e.g. Alexnet, Resnet) (Krizhevsky et al., 2017). While powerful, CNNs assume that images have local spatial correlation. In other words, this means that pixels closer to each should be more closely related than pixels farther away (O'Shea and Nash, 2015 preprint). As a result, CNNs are suited to identifying localized objects (e.g. features) within an image, but they are less suited to ‘comprehend’ entire images holistically or the interactions between various subsections of a single image. Indeed, these tools were recently applied to identify breathing behavior in leatherback turtles *Dermochelys coriacea* in the North Atlantic (Rogers et al., 2024) and while these models successfully identified almost most breathing frames in many deployments, in others, the accuracy dropped to almost 50%. The authors also did not report on how many non-breathing frames were incorrectly labeled as breathing by the model or how effectively the labeled frames could be converted into a biologically relevant metric, such as breathing rate.

To address the short-comings of CNN for image analysis, a tool called Vision Transformer (ViT) was developed with the capacity for global image comprehension (e.g. it does not assume that pixels closer to each other are more closely related than pixels father away) but at the cost of higher computational and data requirements (Dosovitskiy et al., 2020 preprint; Vaswani et al., 2017). In brief, ViT divides each image into smaller sub-sections before assessing how each subsection is related to the others. In the case of identifying sea turtle breathing behavior from animal-borne camera footage, this tool could theoretically identify multiple key features that could help indicate whether a turtle is breathing from both the turtle itself (e.g. the tilt of a turtle's head) and the background (e.g. whether the camera itself is inside or outside the water). ViT also has another benefit over CNNs in that it can create ‘attention maps’ that help establish long-distance connections across the image and illustrate what location on each image the model is using to make predictions, removing some of the ‘black box’ dynamics of many machine-learning tools.

Another potential issue in the use of machine-learning tools for analyzing data from animal-borne cameras is that image processing and feature extraction algorithms often require annotated datasets exceeding millions of images. If the time spent labeling such datasets exceeds the potential time savings from the use of the machine-learning tool, this mitigates the benefits of these tools. Nevertheless, this issue could be potentially alleviated through the use of transfer learning, where a model is trained on an ancillary task where typically a lot of data are available before being fine-tuned to tackle the primary task. This works because the first dataset allows the model to learn how to extract general features, such as edges and contours, whereas the second dataset allows the model to focus on more specific features that are of key interest for addressing the primary task. At present, the most commonly used training dataset for transfer learning is ImageNet, which contains over 14 million images that are categorized into 21,000 unique classes (Deng et al., 2009).

Here, we assessed the accuracy of typical image-processing, feature-extraction and transfer-learning algorithms to identify breaths from animal-borne camera footage on green sea turtles *Chelonia mydas* in The Bahamas. Green turtles, while listed as ‘least concern’ under the International Union for Conservation of Nature (IUCN), are still threated in many areas worldwide (Wallace et al., 2025) and protected via the Convention on International Trade in Endangered Species of Wild Fauna and Flora (CITES). Thus, new knowledge gained from automation of image processing may contribute positively to conservation priorities. Our specific objectives were to (i) compare the efficacy of using Resnet-50 and ViT models, supported by transfer-learning tools, to identify breathing behavior from video footage and then (ii) compare how breathing events (continual series of breathing frames) identified via these models compare with those identified manually.

## MATERIALS AND METHODS

### Study sites and camera deployment

**Fig. 1.**
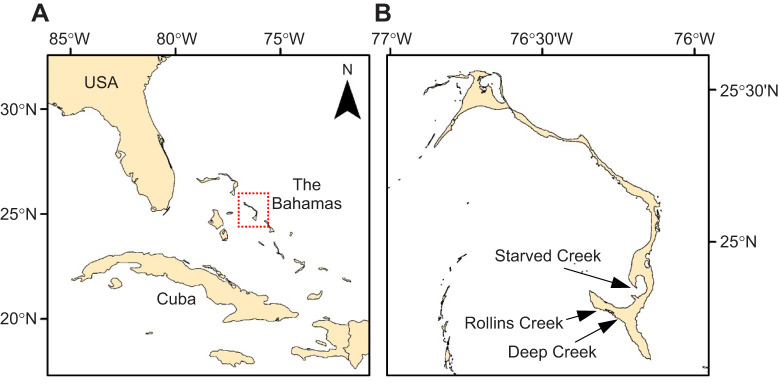
**Map showing the study sites.** The dotted red square illustrates the location of the map on the right.

Juvenile green sea turtles, *Chelonia mydas* (Linnaeus 1758) are common residents of mangrove creeks throughout the island of Eleuthera in The Bahamas (Fig. 1; Robinson et al., 2020; Mills et al., 2023), which is where this study was conducted. Specifically, we sampled turtles found in three mangrove creeks: Rollins Creek, Deep Creek and Starved Creek. The creeks were generally shallow (<5 m depth), with a mix of sandy, muddy and rocky substrates. Beds of turtle grass, *Thalassia testudinum*, and various algal species were present at each location.

Between September 2018 and February 2020, we captured juvenile green turtles via ‘rodeo’. In short, turtles were followed via boat until we were close enough to deploy a snorkeler to capture the animal by hand. Once brought back to the boat, turtles with a straight carapace length >30 cm and without external injuries were selected for the deployment of an animal-borne camera (hereafter referred to as a TurtleCam), with both the methodology and details of the TurtleCam setup described in Robinson et al. (2024). The TurtleCams were programmed to record at 30 frames s^−1^ and resolution of 1920×1080 pixels, and typically recorded 3–4 h of footage based on battery life before the camera released from the carapace and was recovered. Research was conducted under permits provided by The Bahamian Department of Marine Resources (#MA&MR/FIS/9).

### Video annotation and dataset preparation

**Fig. 2.**
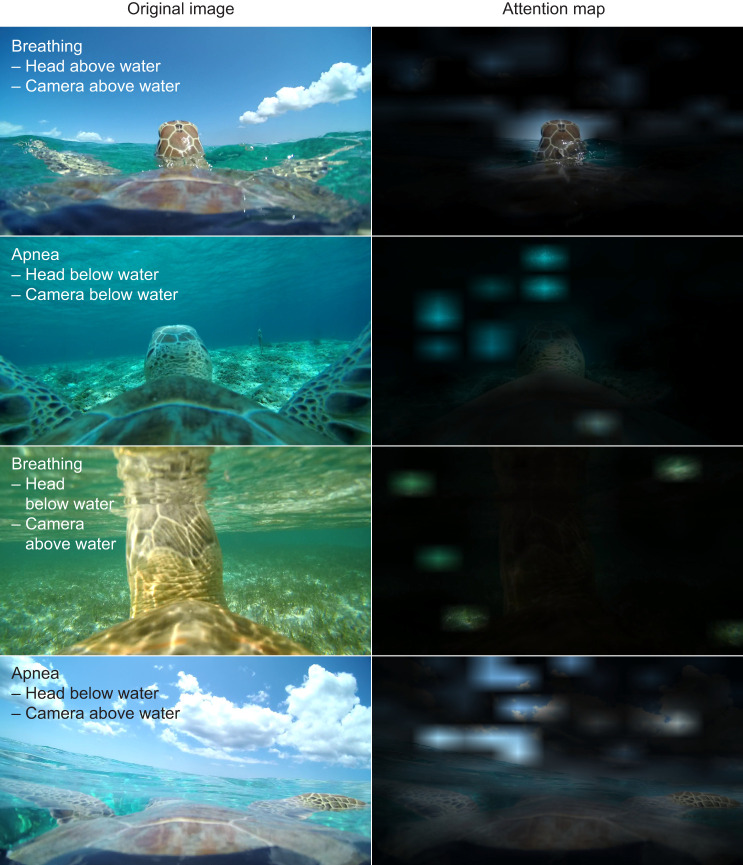
**Example frames of apnea and breathing as used in the training dataset.** We defined breathing as any frame when the turtle's head was above the surface of the water and apnea as any frame when the turtle's head was below the surface of the water. The designations remained accurate regardless of whether the camera was also above or below the water. On the right, we provide attention maps provided by the Vision Transformer (ViT) that visualize the subsections of each image that the model was using to generate predictions.

From a total of 67 successful TurtleCam deployments (for full details, see Robinson et al., 2024), we randomly selected 10 min sections from 18 different deployments on unique green turtles. This resulted in a total of 324,000 frames for this analysis. Each frame was labeled manually by the authors (N.J.R. and L.M.) using VGG Image Annotator (VIA) to identify when turtles were breathing or in apnea (i.e. not breathing). We defined breathing as any moment when the turtle's mouth and nostrils were above the surface of the water (Fig. 2). Furthermore, as the number of breathing frames was only 7460 (2% of the entire dataset) whereas the number of apnea frames was 316,540 (98% of the entire dataset), we dealt with this large class imbalance by using all 7460 breathing frames and then randomly selected 7460 frames from all the apnea frames to use as a training dataset. Finally, we hereafter refer to any continual series of frames identified as breathing as a ‘breathing event’. While each manually labeled breathing event was typically short (less than 60 frames per 2 s), we made the assumption that a single breathing event was synonymous with a single breath.

### Baseline model architecture and training details

We separately used and assessed both a Convolutional Resnet-50 (He et al., 2015 preprint) and a base ViT with patch sizes of 16 (Vaswani et al., 2017) that were already pre-trained on the ImageNet classification task (Deng et al., 2009). The ResNet50 is a deep CNN with 50 layers, designed using residual blocks to mitigate vanishing gradients. Each residual block contains convolutional layers along with normalization and the ReLU activation, and skip connections that enable gradients to flow directly through the network during training. The ViT applies the transformer architecture, originally developed for Natural Language Processing, to images. It splits an image into fixed size patches, which are then processed by the Transformer encoder layer. These encoder layers consist of the Attention mechanism, which learns the pairwise relationships between patches, followed by feedforward projections to process per-patch information.

We took these pre-trained models and used Cross-Entropy Loss to train them on the apnea/breathing TurtleCam frames. The original TurtleCam footage was recorded at 1920×1080 pixel resolution, while the ViT/Resnet-50 required images of 224×224 pixels. As such, images were downsized via interpolation to the necessary resolution before model processing. To promote model generalization and robustness, 50% of images went through image transformations (e.g. flipping the image on the horizontal axis). All models were also trained with a batch size of 128, a learning rate of 0.0002 and the AdamW optimizer with a linear learning rate schedule. The Resnet-50 was trained first for 3 epochs but due to initially poor results and lack of convergence, the model was run a second time until convergence was achieved after 10 epochs (Fig. S1). The ViT was only trained once for 3 epochs as convergence was reached by the first epoch (Fig. S1). We define an epoch as a full pass through of the dataset. Once the training loss begins to reach convergence (e.g. it plateaus), it is likely to start overfitting to the training dataset and so the ViT model was not also run with 10 epochs. The hardware utilized for training was 2×A6000 GPUs, and we leveraged the Huggingface Platform for model and training implementations. The ResNet50 model has approximately 25 million parameters where ViT has 86 million. Each model took less than an hour to process on the aforementioned hardware. Full models are available at https://huggingface.co/priyammaz/TurtleCamClassifier and the data are available at https://huggingface.co/datasets/priyammaz/TurtleCamFrames.

### Model prediction post-processing

As we defined a dive as any period between breathing events, either a single or a series of incorrectly labeled frames could lead to us counting one more dive per section of video and this problem will continue to increase with each mislabeled frame. To mitigate this issue, we assigned each apnea frame a value of 0 and each breathing frame a value of 1. Next, we calculated the running mean per six sequential frames (equivalent to 0.2 s of footage) and then labeled all those frames as either apnea or breathing if the running mean was ≤0.5 or >0.5, respectively. Next, we deleted three frames from the beginning and end of each dive to correct mis-labeling due to the use of the weighted averaging method. Finally, we removed any continual series of breathing frames of fewer than 15 frames in total (0.5 s) as we manually determined that the shortest breathing event from the manually labeled data was 17 frames (Fig. S2). An example of the smoothing process is shown in Fig. S3.

### Model evaluation

For model evaluation, we used the post-processed output (see previous section) and applied a ‘leave one out’ approach whereby the model was trained on 17 of the 18 videos and then assessed via its capacity to label the remaining video. This method was repeated to assess the capacity of the model to label each of the 18 labeled videos. We assessed the capacity of the model after post-processing to distinguish between apnea and breathing frame using the following metrics. (1) Accuracy: proportion of all correctly identified frames (true-positive or true-negative), whether apnea or breathing. (2) Precision: the proportion of all the true-positive breathing frames divided by the number of both true-positive and false-positive frames. (3) Recall: the ratio of correctly labeled breathing frames to the sum of correctly labeled breathing and apnea frames. (4) F1 score: the harmonic mean of precision and recall. F1 Score was useful when seeking a balance between high precision and high recall, as it penalizes extreme negative values of either component. Finally, the ViT model was also used to create Attention Maps, which are essentially ‘heat maps’ that visualize which sections of each image the model considers most relevant to the current task (Dosovitskiy et al., 2020 preprint).

To assess the suitability of the model output for estimating a biologically relevant metric, we calculated the total number of breathing events (defined as any continual series of breathing frames) identified manually to those identified by each model. We also calculated the mean difference (after converting all differences to positive numbers) in the manually identified number of dives (defined as the period between breathing events) compared with the predicted dives. Finally, we divided this number by the number of manually identified breaths to indicate how many breaths were incorrectly identified relative to each manually identified breath. Hereafter, we refer to this as the ratio of correctly to incorrectly identified breaths. Finally, we also used the capacity of the ViT model to create Attention Maps that visualize the subsections of each image that it is using to generate predictions (Fig. 2), to assess where the focus of the model was directed during both correct and incorrect labels.

## RESULTS AND DISCUSSION

### Model performance

Accuracy is the proportion of breathing frames that were correctly labeled by the model. The mean accuracy increased from 86.6% for Resnet50 (3 epochs) to 95.2% for Resnet50 (10 epochs) to 97.2% for ViT (3 epochs) models alongside a decrease in the range in accuracies of individual models from 49.2–98.2% to 84.0–99.8% to 89.3–99.1%, respectively (Table 1; Tables S1, S2 and S3). While appearing promising, because of the class imbalance in the videos (i.e. 98% of frames were of apnea), a model that exclusively predicted apnea frames would still have an accuracy of 98%. Nevertheless, the accuracy of the ResNet50 (3 epochs) model, which ranged from 49.2% to 98.2%, was similar to that reported from the use of other CNN models to assess breathing rates of leatherback turtles from animal-borne camera footage (Rogers et al., 2024) or in green turtles using TDRs (Francke et al., 2013). These values were, however, far exceeded by the ResNet50 (10 epochs) and the ViT model, for which the lowest accuracy per deployment was 84.0% and 89.3% respectively.

**Table 1.** Aggregate Measures of Accuracy, Precision, Recall, F1 score and the ratio of correctly to incorrectly identified breaths across all videos with leave-one-out prediction results

Model architecture	Accuracy (%)	Precision (%)	Recall (%)	F1 (%)	Ratio of correctly to incorrectly identified breaths
Resnet50–3 epochs	86.6 [49.2, 98.2]	19.6 [3.5, 46.3]	100 [99.7, 100]	55.0 [6.9, 63.3]	2.48
Resnet50–10 epochs	95.2 [84.0, 99.8]	47.6 [14.7, 82.3]	99.9 [99.4, 100]	60.3 [25.6, 90.3]	0.17
ViT – 3 epochs	97.2 [89.3, 99.1]	63.3 [17.9, 98.1]	99.1 [92.8, 100]	72.7 [30.4, 95.4]	0.10

Data presented are means with range in brackets.

Precision compares the number of frames correctly labeled as breathing with all frames labeled as breathing, both correct or incorrect. As such, precision can be a flawed metric for evaluating a dataset like ours with distinct class imbalances, as a small percentage of incorrectly labeled apnea frames could significantly reduce the model's precision. It was therefore somewhat predictable that precision was low in all models. Nevertheless, the mean precision increased from 19.6% in Resnet50 (3 epochs) to 47.6% in Resnet50 (10 epochs), and then to 63.4% in ViT (3 epochs) models even though the range in precision of individual models remained very high at 3.5–46.3%, 14.7–82.3% and 17.9–98.1%, respectively (Table 1; Tables S1, S2 and S3).

Recall is the proportion of manually identified breathing frames that were classified correctly as breathing frames by the model, and should be robust to the previously mentioned issue of class imbalance. Encouragingly, the mean recall for all models ranged from 99.1% to 100.0% and the recall of any individual model was always above 92.8%. Unlike in the two previous metrics, Resnet50 (3 epochs) was the highest performing model and ViT (3 epochs) model was the lowest (Table 1; Tables S1, S2 and S3). It should be noted that the Recall of all models (mean range: 99.1–100.0%) WAS notably higher than the Recall values of 0.62–0.63 achieved in a similar study but using accelerometry data instead of animal-borne camera footage, when estimating breathing behavior from loggerhead turtles *Caretta caretta* (Agabiti et al., 2026). In other words, almost all breathing frames were classified correctly in our study and the percentage of apnea frames incorrectly labeled as breathing was also relatively small (3.9%). The current ResNet50 or ViT models may therefore not yet be sufficient to automatically estimate breathing rate, but they represent a notable improvement over the use of previous CNN models (e.g. Rogers et al., 2024) as well as many TDR and accelerometry studies for estimating breathing rates (e.g. Jeantet et al., 2024; Agabiti et al., 2026). Moreover, the almost perfect Recall of the ViT models indicates that these tools can rapidly highlight all potential frames that could then be reviewed manually to confirm whether they show breathing or not. The use of such a tool could still massively reduce the number of frames that need to be reviewed when manually labeling breathing versus apnea frames, in turn saving researchers considerable effort and time.

The F1 score combines both Precision and Recall metrics, and typically F1 scores over 70% are ‘good’ (Naidu et al., 2023). As Recall was almost 100% in all models, the F1 score should be primarily influenced by variation in Precision. Mean F1 scores increased from 55.0% in Resnet50 (3 epochs) to 60.3% in Resnet50 (10 epochs), and then to 72.7% in ViT (3 epochs) (Table 1; Tables S1, S2 and S3). The fact that the ViT model outperformed the Resnet model even when the latter was trained over a higher number of epochs highlights the value of using AI-tools with global image comprehension for identifying behaviors in animal-borne camera footage. These F1 values of the ViT model were also comparable to other machine-learning based models that have been used to identify behaviors, such as feeding and resting (but not breathing), using accelerometry data from both sea turtles (Jeantet et al., 2024) and seabirds (Otsuka et al., 2024). This suggests that ViT models can be just as effective at the automated behavioral identification from video footage as has been previously achieved using arguable less complex data inputs (e.g. numerical dive or accelerometry data).

### Identifying individual breathing events

**Fig. 3.**
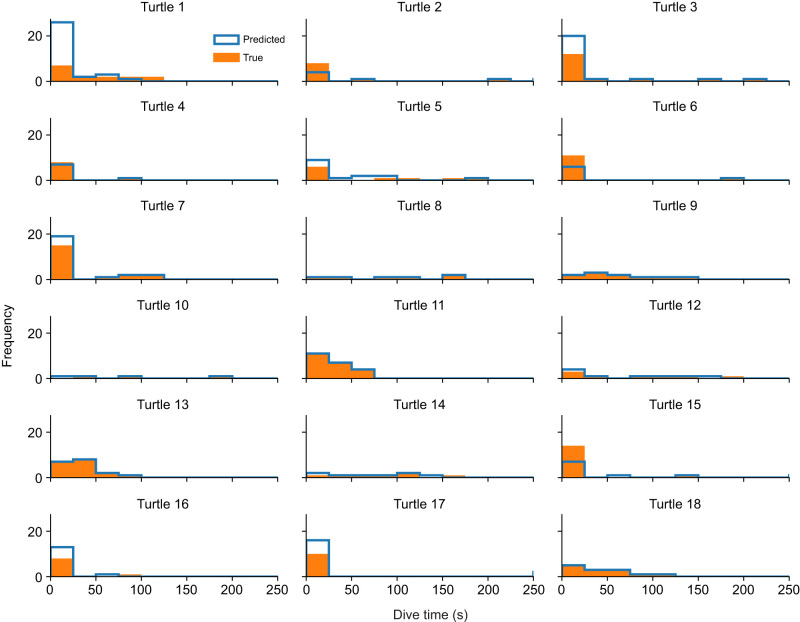
Manually identified time between breaths compared with the ViT (3 epochs) model-predicted time between breaths.

The Resnet50 (3 epochs) never correctly predicted the number of breathing events per video, overestimating in 16 videos and underestimating in the other two. Specifically, for every correctly identified breathing event there were 2.48 incorrectly identified. The Resnet50 (10 epochs) exhibited a marked improvement over the Resnet50 (3 epochs) and correctly predicted the number of breathing events in eight videos while also overestimating in six videos and underestimating in four. Specifically, for every correctly identified breathing event there were 0.17 incorrectly identified. Finally, the ViT (3 epochs) continued to improve upon both previous models and correctly predicted the manually identified number of breathing events in 12 of 18 videos while still overestimating in four videos and underestimating in two. Specifically, for every correctly identified breathing event there were 0.10 incorrectly identified. By comparing the time between breaths for each video (Fig. 3), the majority of errors in the ViT (3 epochs) model were observed when the time between breathing events was <25 s or >125 s.

### Attention maps

**Fig. 4.**
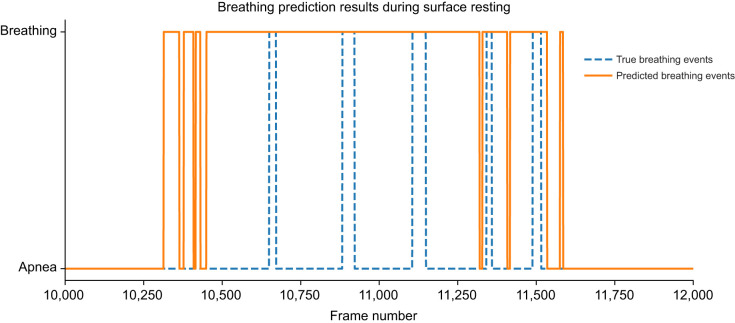
**An example output from the ViT model where the TurtleCam remained above the surface of the water while a turtle was conducting several breathing events.** In this instance, the model output (orange line) struggled to correctly identify each breathing event (dashed blue lines).

Reviewing the attention maps generated by the ViT model shows that on correctly identified breathing events, the attention was typically focused on the sea turtle's head rising above the surface of the water, which matches how we defined breathing in ‘Video annotation and dataset preparation’, above (Fig. 2). Similarly, in incorrectly identified apnea frames, we observed that the head was the key focal point in the attention maps, whereas in incorrectly predicted breathing frames, the focus was largely distributed across the background of the image. This suggests that the model has ‘learned’ that the key feature to be observed when discriminating between apnea and breathing is the head. Nevertheless, the primary sources of mislabeled breathing frames appear to result from two key events. (1) When the turtle came to the surface and took multiple breaths consecutively without actively swimming/diving away from the surface. In such instances, the camera often remained above the water's surface (Fig. 2) and instead of identifying each breath, the model rapidly switched back and forth between breathing and apnea, vastly over-estimating the number of breathing events while the turtle remained at the surface (Fig. 4). (2) When the turtle surfaced for a breath, but the camera itself did not break the surface of the water (Fig. 2). When this occurred, the model often did not identify a breathing event and thus overestimated the time between breathing events. Both of these issues indicate that the model was imprecise in discriminating between when the turtle was breathing or when the camera itself was above the water's surface but the turtle's head remained below the surface. One potential solution would therefore be to first adjust the model to identify surfacing events (defined as periods of inactivity at the surface that may be punctuated by multiple breathing events) instead of breathing events. However, this may reduce the biological insights gained from the animal-borne cameras when trying to assess breathing and diving patterns, and would not present a significant advance over what is already achievable using depth sensors. Alternatively, we could potentially increase the height of the camera and thus ensure that it broke the water's surface upon every surfacing event. While the first solution would be an interesting angle for further research, we caution against the second option as it would likely increase the hydrodynamic drag associated with the bio-logging device (e.g. Jones et al., 2013) and such a trade-off to improve analysis times would not be justifiable.

### Future model improvements

While we used a running mean (described in ‘Model prediction post-processing’, above) to address the issues associated with a single mislabeled frame, a more refined solution could be to incorporate a temporal ‘memory’ into the model. The current model produces a prediction for every frame individually, but has no context for preceding and future frames, which is a major limitation as most behaviors we are interested in span multiple sequential frames of video footage. As discussed earlier, the ViT model interprets images via the learned relationships across the different subsections, although these subsections are for a single image. More advanced architectures, such as the VideoMAE (Tong et al., 2022) can learn relationships across the subsections not only of a single image but also between all subsections of an entire sequence of images. By doing this, the model can learn both the spatial and temporal dynamics to produce its predictions.

Another way to potentially improve any machine-learning algorithm is to provide ever larger training datasets. In our model, we used transfer-learning via ImageNet (Deng et al., 2009; Krizhevsky et al., 2017) to provide a source of images to pre-train our models on, and as these models continually grow, their utility for image processing will simultaneously increase. Moreover, as the use of animal-borne cameras on sea turtles continues to increase (e.g. Mullaney et al., 2024; Robinson et al., 2024; Manning et al., 2025), the amount of highly relevant training footage will also increase. As the sea turtle species, camera quality and environmental conditions (e.g. water quality, benthic substrate, etc.) will differ in many of these training datasets, the resulting model will grow increasingly robust until a point where hopefully it can be applied to novel sea turtle species in novel habitats while still retaining a high level of performance.

### Conclusion

We illustrated that ViT models can serve as useful tools for identifying breathing behavior from animal-borne camera footage from sea turtles, with improved reliability beyond CNN tools or inferences derived from TDRs and/or accelerometers. While the ViT model still has some limitations when determining biologically relevant metrics, such as breathing rates, its high Recall values show that ViT models can already massively reduce the number of frames that researchers need to annotate. The next steps should therefore focus on how ViT models perform for identifying breathing behavior in other air-breathing marine species and more visually complex behaviors such as foraging and socializing. Moreover, we envisage that the strength of image processing tools will continue to advance in the coming years alongside a mix of technological innovation and, if publicly shared, an ever-increasing abundance of potential training datasets.

## Supplementary Material

10.1242/jexbio.251688_sup1 Supplementary information
