# Supplementary material for: Classifying behaviors from animal-borne cameras using machine learning: automated identification of breathing events in sea turtles
Source: J Exp Biol. 2026 Jun 10;229(11):jeb251688. doi: 10.1242/jeb.251688 (PMC13286345; doi:10.1242/jeb.251688)
Supplement: Supplementary information [file jexbio-229-251688-s1.pdf]

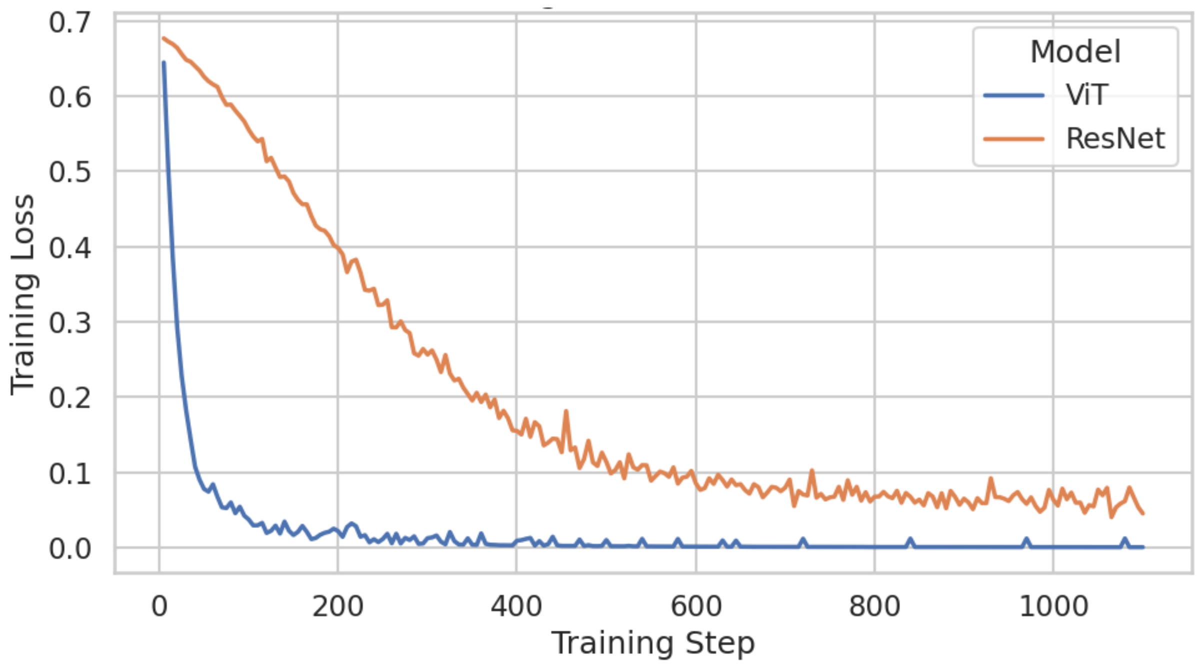

Fig. S1. Training loss for the ResNet and ViT models. Each epoch is 100 steps of training. The ResNet model plateaus around 10 epochs, and so we tested the model at both 3 and 10 epochs. The ViT plateaued much quicker and thus was only tested with 3 epochs.

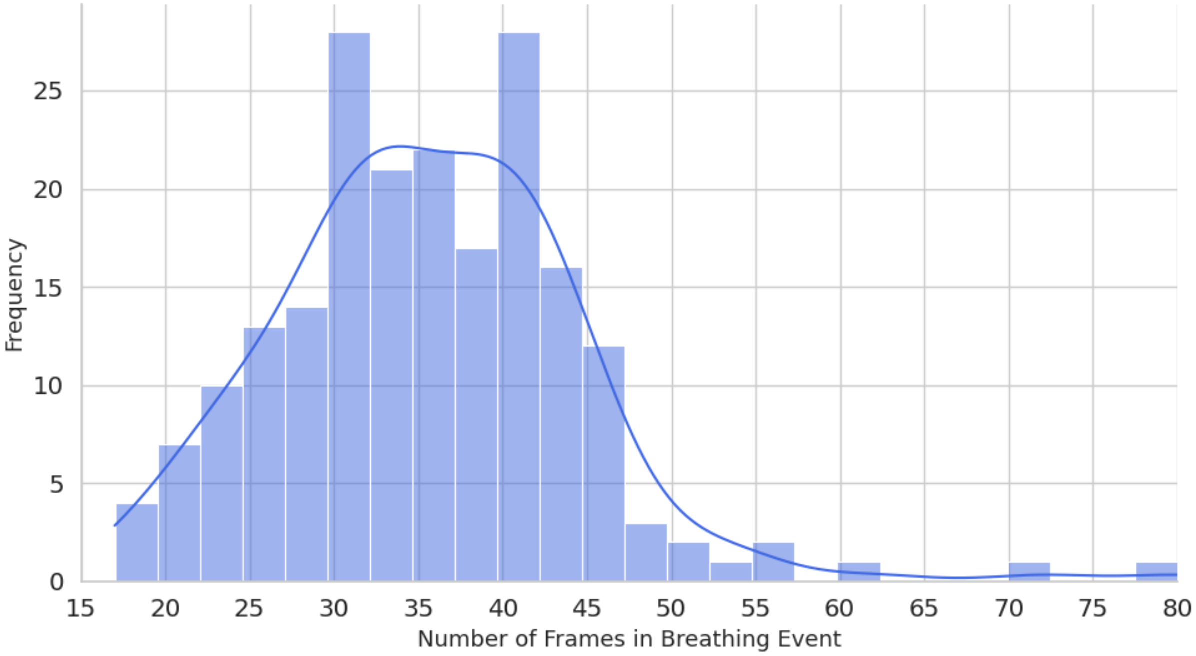

Fig. S2. A histogram showing the number of frames in each manually labelled breathing event. As no manually labelled breathing events were shorter than 17 frames, we removed any continual series of breathing frames less than 15 frames in total from the modelled data.

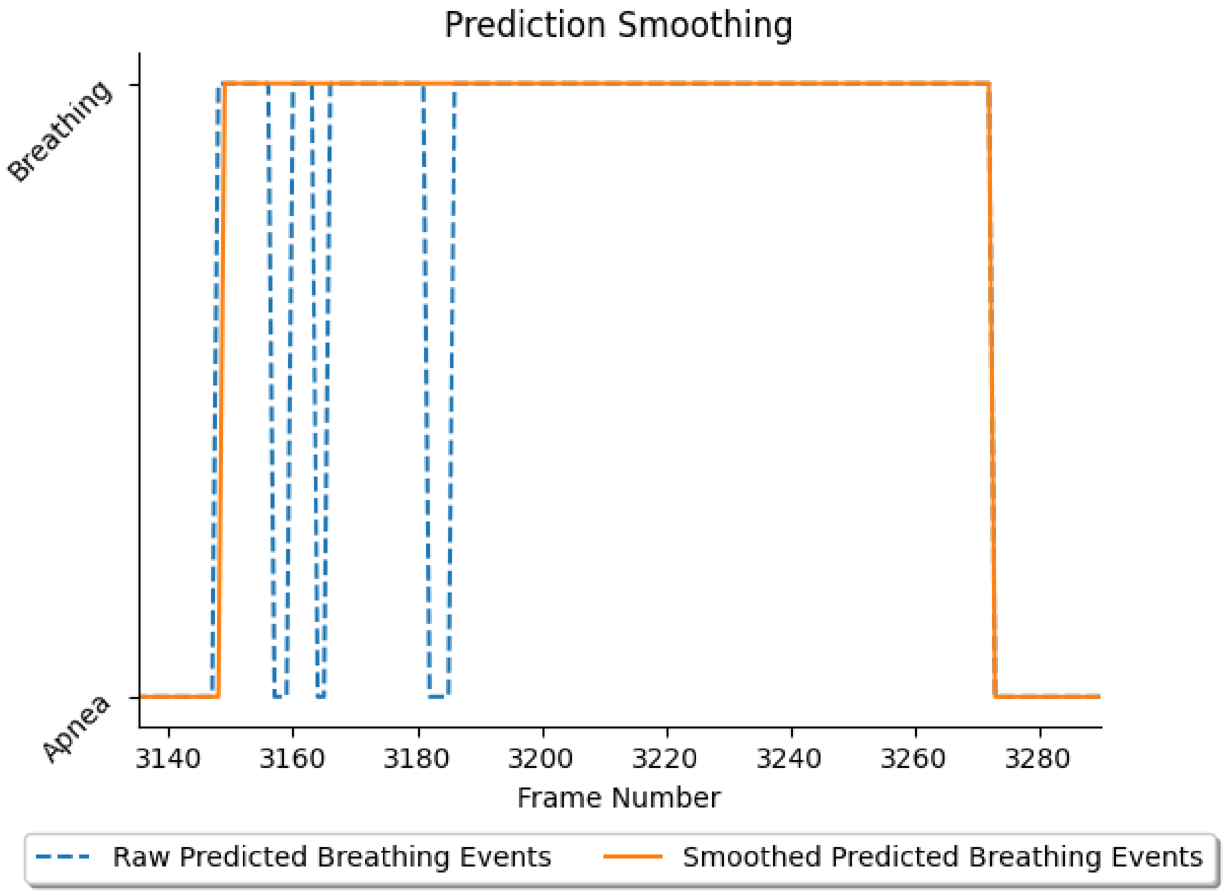

Fig. S3. An example of output from the Resnet-50 model for identifying breathing events before applying the smoothing algorithm (a running-average over 6 frames) as described in section 2.4.

Journal of Experimental Biology • Supplementary information

Table S1. Accuracy, Precision, Recall, F1 Score and Manually Identified vs Predicted Number of Breathing events for Leave-One-Out prediction results from the Resnet50 (3 epochs) model.

Video Name	Accuracy	Precision	Recall	F1	# True/Pred of Breathing Events
TurtleCam12_Video17	85.9%	10.2%	100%	18.5%	11/22
TurtleCam13_Video5	88.3%	14.8%	100%	25.9%	15/30
TurtleCam14_Video15	49.2%	3.5%	100%	6.9%	9/118
TurtleCam15_Video15	78.8%	10.6%	100%	19.2%	14/27
TurtleCam19_Video11	81.7%	14.7%	100%	25.6%	15/13
TurtleCam20_Video18	83.6%	8.3%	100%	15.4%	9/38
TurtleCam24_Video9	72.6%	7.8%	100%	14.5%	12/76
TurtleCam25_Video15	90.6%	18.8%	99.7%	31.8%	9/36
TurtleCam35_Video4	93.1%	26.9%	100%	42.4%	12/5
TurtleCam36_Video6	84.9%	21.5%	100%	35.3%	19/27
TurtleCam37_Video17	93.6%	15.0%	100%	26.1%	5/19
TurtleCam41_Video16	96.7%	37.8%	100%	54.9%	9/18
TurtleCam6_Video20	88.6%	5.2%	100%	9.8%	3/28
TurtleCam16_Video7	98.2%	35.6%	100%	52.5%	6/8
TurtleCam7_Video2	94.1%	46.3%	100%	63.3%	21/25
TurtleCam8_Video8	93.7%	16.2%	100%	28.0%	7/27
TurtleCam9_Video2	93.2%	32.4%	100%	48.9%	17/26
TurtleCam23_Video12	91.1%	18.7%	100%	31.4%	11/22

Journal of Experimental Biology • Supplementary information

Table S2. Accuracy, Precision, Recall, F1 Score and True vs Predicted Number of Breathing events for Leave-One-Out prediction results from the Resnet50 (10 epochs) model.

Video Name	Accuracy	Precision	Recall	F1	# True/Pred # of Breathing Events
TurtleCam12_Video17	90.7%	14.7%	100%	25.6%	11/6
TurtleCam13_Video5	95.5%	31.2%	100%	47.6%	15/17
TurtleCam14_Video15	90.0%	15.8%	99.7%	27.3%	9/7
TurtleCam15_Video15	90.8%	21.5%	100%	35.4%	14/21
TurtleCam19_Video11	84.0%	16.5%	100%	28.2%	15/8
TurtleCam20_Video18	91.7%	15.2%	100%	26.4%	9/11
TurtleCam24_Video9	99.2%	75.1%	100%	85.8%	12/12
TurtleCam25_Video15	98.2%	55.9%	99.4%	71.1%	9/13
TurtleCam35_Video4	94.4%	31.5%	100%	47.9%	12/6
TurtleCam36_Video6	88.1%	25.8%	100%	41.0%	19/20
TurtleCam37_Video17	99.7%	78.7%	100%	88.0%	5/5
TurtleCam41_Video16	98.9%	64.9%	100%	78.7%	9/9
TurtleCam6_Video20	99.8%	82.3%	100%	90.3%	3/3
TurtleCam16_Video7	99.5%	68.8%	100%	81.6%	6/6
TurtleCam7_Video2	98.2%	74.0%	99.8%	85.0%	21/21
TurtleCam8_Video8	99.5%	68.7%	100%	81.5%	7/8
TurtleCam9_Video2	98.6%	70.4%	99.8%	82.6%	17/17
TurtleCam23_Video12	97.5%	45.7%	100%	62.7%	11/13

Journal of Experimental Biology • Supplementary information

Table S3. Accuracy, Precision, Recall, F1 Score and True vs Predicted Number of Breathing events for Leave-One-Out prediction results from the Vision Transformer (3 epochs) model.

Video Name	Accuracy	Precision	Recall	F1	# True/Pred # of Breathing Events
TurtleCam12_Video17	94.0%	21.0%	100%	34.6%	11/11
TurtleCam13_Video5	98.3%	55.0%	100%	70.9%	15/15
TurtleCam14_Video15	91.0%	17.9%	100%	30.4%	9/8
TurtleCam15_Video15	99.8%	92.8%	99.7%	91.7%	14/14
TurtleCam19_Video11	89.3%	22.7%	100%	37.1%	15/25
TurtleCam20_Video18	94.7%	22.2%	100%	36.5%	9/12
TurtleCam24_Video9	99.1%	73.5%	100%	84.7%	12/12
TurtleCam25_Video15	99.0%	77.2%	99.4%	86.9%	9/10
TurtleCam35_Video4	95.8%	37.7%	100%	54.9%	12/9
TurtleCam36_Video6	91.8%	33.7%	100%	50.5%	19/25
TurtleCam37_Video17	99.8%	86.0%	100%	92.4%	5/5
TurtleCam41_Video16	99.6%	80.9%	100%	89.4%	9/9
TurtleCam6_Video20	99.9%	98.1%	92.8%	95.4%	3/3
TurtleCam16_Video7	99.8%	84.2%	100%	91.4%	6/6
TurtleCam7_Video2	98.7%	80.4%	99.3%	88.8%	21/21
TurtleCam8_Video8	99.8%	87.9%	100%	93.5%	7/7
TurtleCam9_Video2	99.6%	93.8%	94.3%	94.0%	17/17
TurtleCam23_Video12	99.3%	75.1%	100%	85.7%	11/11

Journal of Experimental Biology • Supplementary information
